# A feasibility study for objective evaluation of visual acuity based on pattern-reversal visual evoked potentials and other related visual parameters with machine learning algorithm

**DOI:** 10.1186/s12886-023-03044-7

**Published:** 2023-06-27

**Authors:** Jian Zheng Chen, Cong Cong Li, Shao Heng Li, Yu Ting Su, Tao Zhang, Yu Sheng Wang, Guo Rui Dou, Tao Chen, Xiao Cheng Wang, Zuo Ming Zhang

**Affiliations:** 1 Ministry-of-Education Key Laboratory of Aerospace Medicine, School of Aerospace Medicine, Air Force Medical University, Xi’an, Shaanxi Province China; 2 Qingdao Special Servicemen Recuperation Center of PLA Navy, Qingdao, Shandong Province China; 3 Department of Ophthalmology, The First Affiliated Hospital, Air Force Medical University, Xi’an, Shaanxi Province China; 4 School of Biomedical Engineering, Air Force Medical University, Xi’an, Shaanxi Province China; 5 Department of Aviation Medicine, The First Affiliated Hospital, Air Force Medical University, Xi’an, Shaanxi Province China

**Keywords:** Visual acuity, Visual-evoked potentials, Refractive error, Aircrew, Machine learning

## Abstract

**Background:**

To develop machine learning models for objectively evaluating visual acuity (VA) based on pattern-reversal visual evoked potentials (PRVEPs) and other related visual parameters.

**Methods:**

Twenty-four volunteers were recruited and forty-eight eyes were divided into four groups of 1.0, 0.8, 0.6, and 0.4 (decimal vision). The relationship between VA, peak time, or amplitude of P100 recorded at 5.7°, 2.6°, 1°, 34′, 15′, and 7′ check sizes were analyzed using repeated-measures analysis of variance. Correlations between VA and P100, contrast sensitivity (CS), refractive error, wavefront aberrations, and visual field were analyzed by rank correlation. Based on meaningful P100 peak time, P100 amplitude, and other related visual parameters, four machine learning algorithms and an ensemble classification algorithm were used to construct objective assessment models for VA. Receiver operating characteristic (ROC) curves were used to compare the efficacy of different models by repeated sampling comparisons and ten-fold cross-validation.

**Results:**

The main effects of P100 peak time and amplitude between different VA and check sizes were statistically significant (all *P* < 0.05). Except amplitude at 2.6° and 5.7°, VA was negatively correlated with peak time and positively correlated with amplitude. The peak time initially shortened with increasing check size and gradually lengthened after the minimum value was reached at 1°. At the 1° check size, there were statistically significant differences when comparing the peak times between the vision groups with each other (all *P* < 0.05), and the amplitudes of the vision reduction groups were significantly lower than that of the 1.0 vision group (all *P* < 0.01). The correlations between peak time, amplitude, and visual acuity were all highest at 1° (*r*_*s*_ = − 0.740, 0.438). VA positively correlated with CS and spherical equivalent (all *P* < 0.001). There was a negative correlation between VA and coma aberrations (*P* < 0.05). For different binarization classifications of VA, the classifier models with the best assessment efficacy all had the mean area under the ROC curves (AUC) above 0.95 for 500 replicate samples and above 0.84 for ten-fold cross-validation.

**Conclusions:**

Machine learning models established by meaning visual parameters related to visual acuity can assist in the objective evaluation of VA.

## Background

Visual acuity (VA) examination, one of the most basic tests for visual function, is divided into subjective and objective examination methods. The subjective ways are simple and easy to perform using various visual acuity charts. However, these methods require a high degree of patient cooperation. Infants, children with cerebral visual impairment, pseudo-blindness and hysterical patients need an objective VA assessment method, a classic common challenge for ophthalmologists. Likewise, this assessment technology is also required for the VA identification of flight personnel.

Pattern-reversal visual-evoked potentials (PRVEPs) are a cluster of bioelectrical signals generated in the visual cortex located in the brain’s occipital lobe after graphic visual stimulus. They are waveforms recorded by regular graphic stimulation of the subject and processed by applying computerized averaging and superimposition techniques. The P100 wave is the first positive wave to appear in PRVEPs. It includes peak time and amplitude evaluation elements, with low variability across subjects and a strong relationship with VA. PRVEPs are mainly applied in patients who are unable or unwilling to complete a subjective VA examination and those with cognitive deficits. They are widely used in the assessment of optic neuropathy [1–3] and functional visual loss [4], in the identification of malingering [5], and in the early diagnosis and prognostication of multiple sclerosis [6–8]. Over the years, several reports have demonstrated that using the visual-evoked potential technique to detect the completeness of visual pathways has been widely recognized in ophthalmology [9, 10]. However, most prior studies on the objective assessment of VA have focus on normal VA or best-corrected VA; there is little information in the literature on the effect of reduced VA caused by refractive error alone on the PRVEPs waveform [10, 11]. When visual acuity was greater than Snellen 20/100, there was a good correlation between subjective VA and PRVEP-estimated VA and dioptric blur would diminish the PRVEP responses to smaller pattern elements more than to larger ones [12]. In addition, it has been shown that the correlation between PRVEP-estimated VA and subjective VA in patients with organic ocular pathology is less than that in subjects with refractive error only [12]. VA is also related to visual parameters such as contrast sensitivity (CS), refractive error, wavefront aberrations, and visual field [13–16]. Therefore, we studied the correlation of these parameters with VA and incorporated them into machine modeling with the P100 of PRVEPs. As a subfield of artificial intelligence, machine learning has been increasingly applied to medical practice since it can extract deeper features from raw data and combine several predictors in a highly interactive manner [17]. In recent years, machine learning algorithms have become more prevalent in the diagnosis and prognostication of many ophthalmic diseases [18–20].

This study focused on the relationship between VA and visual parameters, including PRVEPs, CS, refractive error, wavefront aberrations, and visual field. We were using machine learning algorithms to explore a feasible objective examination and assessment method for VA to provide theoretical support and an experimental basis for improving the quality of vision assessment for special operation staff, such as pilots.

## Methods and subjects

### Subjects

Twenty-four male subjects from Air Force Medical University were recruited; their ages ranged from 20 to 35 (mean 27.75 ± 4.33) years. The inclusion criteria for selecting participants were as follows: spherical degree (D) − 2.00 D ~ + 2.50 D, cylindrical degree − 1.00 D ~ + 1.00 D, and best-corrected VA ≥ 1.0 (decimal vision). The exclusion criteria were a history of refractive surgery, central nervous system disease, glaucoma, diabetic retinopathy, and other organic ocular diseases. The experiments were conducted in the same laboratory under the same luminance environment, and all subjects rested and adapted to the light environment for 10 min before the examination. An automated, computerized refractive examiner (KR-8100PA, Topcon, Japan) was used to detect the subjects’ refractive condition. First, the refractive error of the subjects was examined. After meeting the criteria, twenty-four subjects were randomly divided into four groups: 1.0/1.0, 0.8/0.8, 0.6/0.6, and 0.4/0.4 (decimal vision). Six subjects and twelve eyes were in each group. Second, subjects were checked for monocular VA at 5 m using a standard logarithmic visual acuity E chart with 96% contrast in a 200 cd/m^2^ luminance lightbox. Finally, the subjects were corrected to the corresponding VA using the subjective insertion method with appropriate lenses.

### Pattern visual evoked potential

The VEP stimulated by reversal checkerboard patterns was recorded using the Visual Electrophysiology System (LS-D1, ChongQing Sunkingdom, China). The field size of the checkerboard stimulus was 375 × 300 mm, the contrast of the checkerboard was 96%, and the average luminance of the screen was 120 cd/m^2^. Each subject sat 1 m from a black-and-white checkerboard monitor in a moderately lit room, with their eyes at the same height as the center of the screen. The reversal rate was two reversals per second. Monocular PRVEPs of both eyes (first the right, then the left) were recorded using gold disc scalp electrodes with an average of 100 stimulations. According to the International Society for Clinical Electrophysiology of Vision standard [21], the active electrode was placed at Oz with the reference electrode at Fz, and the ground electrode at the right earlobe, all with inter-electrode impedance < 5 kΩ. Six sizes of checkerboard patterns (5.7°, 2.6°, 1°, 34′, 15′ and 7′ check sizes) were tested in each eye, with 1 to 2 min of rest between each examination with the eyes closed. Throughout the test, the examiner closely monitored the participants’ fixation on a red marker at the center of the screen. Each eye was examined at least two times at each check size, and similar graphs were considered reliable. The entire PRVEPs examination was 20 ~ 30 min for each subject.

### Other visual parameters

CS examinations were performed by MetroVision (MonPack one, Perenchies, France) under photopic conditions (80 cd/m^2^) while the patient was sitting 2 m away from the screen, with one eye of the corrective lenses. Spatial frequencies of 0.6, 1.1, 2.2, 3.4, 7.1, and 14.2 cycle per degree (cpd) were tested. The refractive error was calculated from the refractive error of the bare eye and the corrective lenses. Spherical equivalent (SE) was calculated using the standard formula (spherical equivalent = spherical power + [cylinder power/2]). Wavefront aberrations for a 4 mm pupil were tested by the OPD-Scan III aberrometer (Nidek, Gamagori, Japan). To avoid the influence of lenses on the examination, we recorded the root-mean-square (RMS) values for the total higher-order aberrations (HoA), total coma aberrations (CA), total trefoil aberrations (TA), and total spherical aberrations (SA) in the naked eye. The visual field was determined using a Humphrey Field Analyzer 750i (Zeiss Humphrey Systems, Dublin, CA). The central 30 − 2 threshold protocol was performed in each eye with the corrective lenses. The absolute values of mean deviation (MD) and pattern standard deviation (PSD) were recorded for statistical analysis.

### Statistical analysis

SPSS version 21.0 (IBM, USA) was used for statistical analysis. Measurement data were expressed as mean ± standard deviation ($\stackrel{-}{x}$± *s*). Kolmogorov-smirnov test was conducted on the data, and the results showed that the data conformed to the normal distribution. Statistical analyses were performed using repeated-measures analysis of variance (ANOVA) and rank correlation with a significance level of 5%. The univariate ANOVA method was used if the sphericity test was satisfied. Otherwise, the multivariate ANOVA method was used. Further two-by-two comparisons were carried out using the least significant difference test. Subjective VA was binarized using 1.0, 0.8, and 0.6 VA as cutoff points, and the mean area under the ROC curves (AUC) was used to compare the performances of different models based on meaningful objective visual parameters.

### Machine learning modeling

Machine learning models are computer programs used to recognize patterns in data or make predictions. The models are created from machine learning algorithms, which are trained using labeled, unlabeled, or mixed data. Different machine learning algorithms are suited to different goals, such as classification or prediction modeling, so data analysts use different algorithms as the basis for different models.

**Fig. 1:**
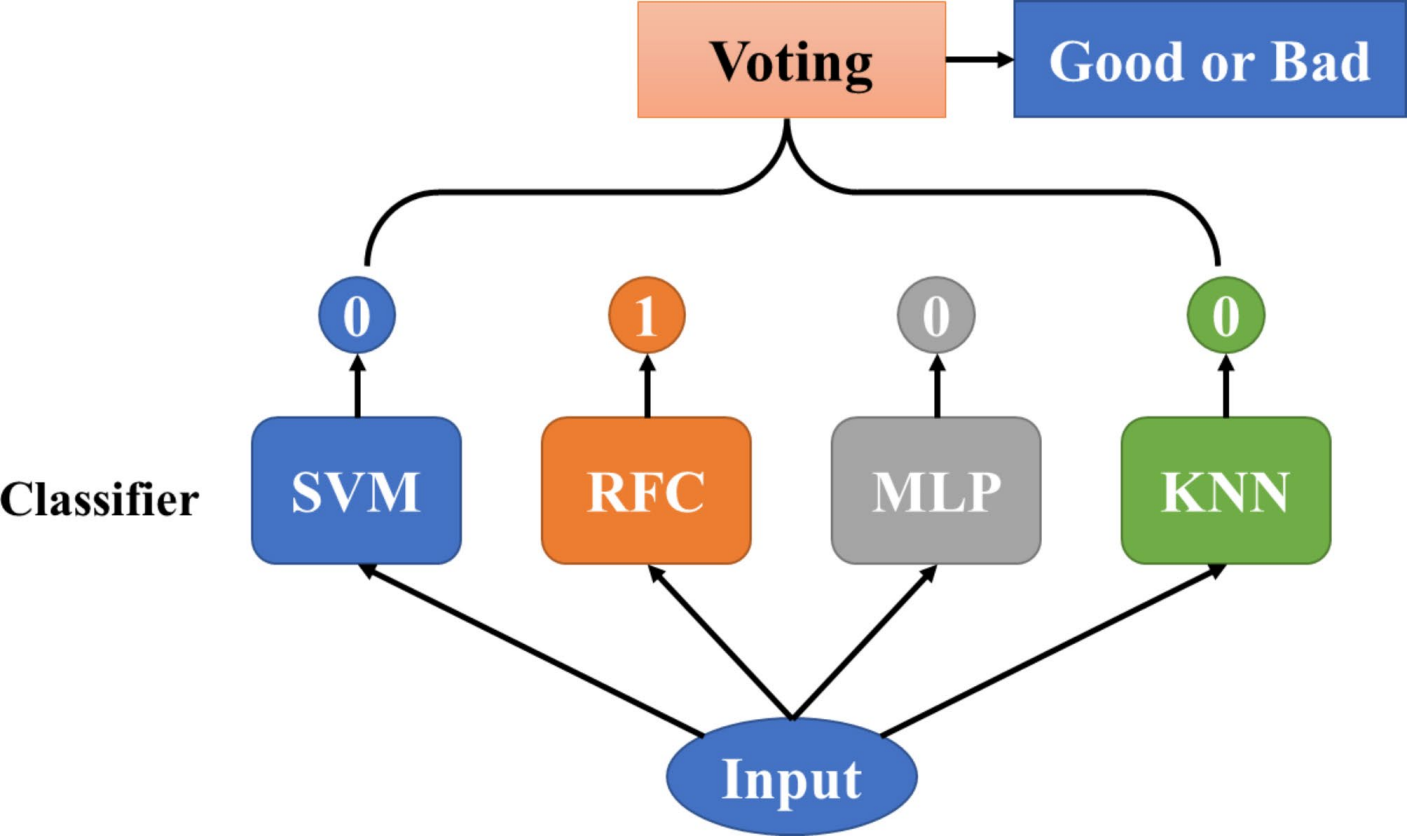
Hybrid classifier based on voting, which utilized support vector machine (SVM), random forest (RF), K-nearest neighbor (KNN), and multilayer perceptron (MLP) as the basic classifiers

Based on the Scikit Learn machine learning algorithm package in Python, the P100 and other visual parameters related to VA were used as input variables, and the binarized VA divided by different VA cutoffs (1.0, 0.8, and 0.6) were used as output variables to construct classification models by support vector machine (SVM), random forest (RF), K-nearest neighbor (KNN), multilayer perceptron (MLP), and Voting. Hybrid Classifier uses a combination of different classifiers to make a common decision. Voting is a common method of classifier ensemble, aggregating the predictions of each basic classifier and using the one with the most votes as the final prediction. Voting has the advantage of achieving better results than individual classifiers by complementing the strengths of each basic classifier. In this study, a voting classifier was constructed as shown in Fig. 1. It utilizes SVM, RF, KNN, and MLP as the basic classifiers to obtain the final prediction based on the voting results. The classification performance of the models was compared. On one hand, 70% of the 48-sample data were selected as the training set and 30% as the test set, and the training test was repeatedly sampled 500 times to compare the mean AUC of each classification model. On the other hand, ten-fold cross-validation was performed on the 48-sample data to compare the accuracy, sensitivity, specificity, and other parameters of different classification models. In the ten-fold cross-validation, the 48-sample data were randomly divided into 10 parts, and when one part was used as the validation set, the other 9 parts were used as the training set, and the same binary classification of “good” and “bad” visual acuity was performed. The 10 samples were cross-trained and validated 10 times, and the results of the ten-fold cross-validation of each model were obtained. The accuracy, sensitivity and specificity of the classification were compared.

## Results

### P100 peak time versus VA at different check sizes

**Fig. 2:**
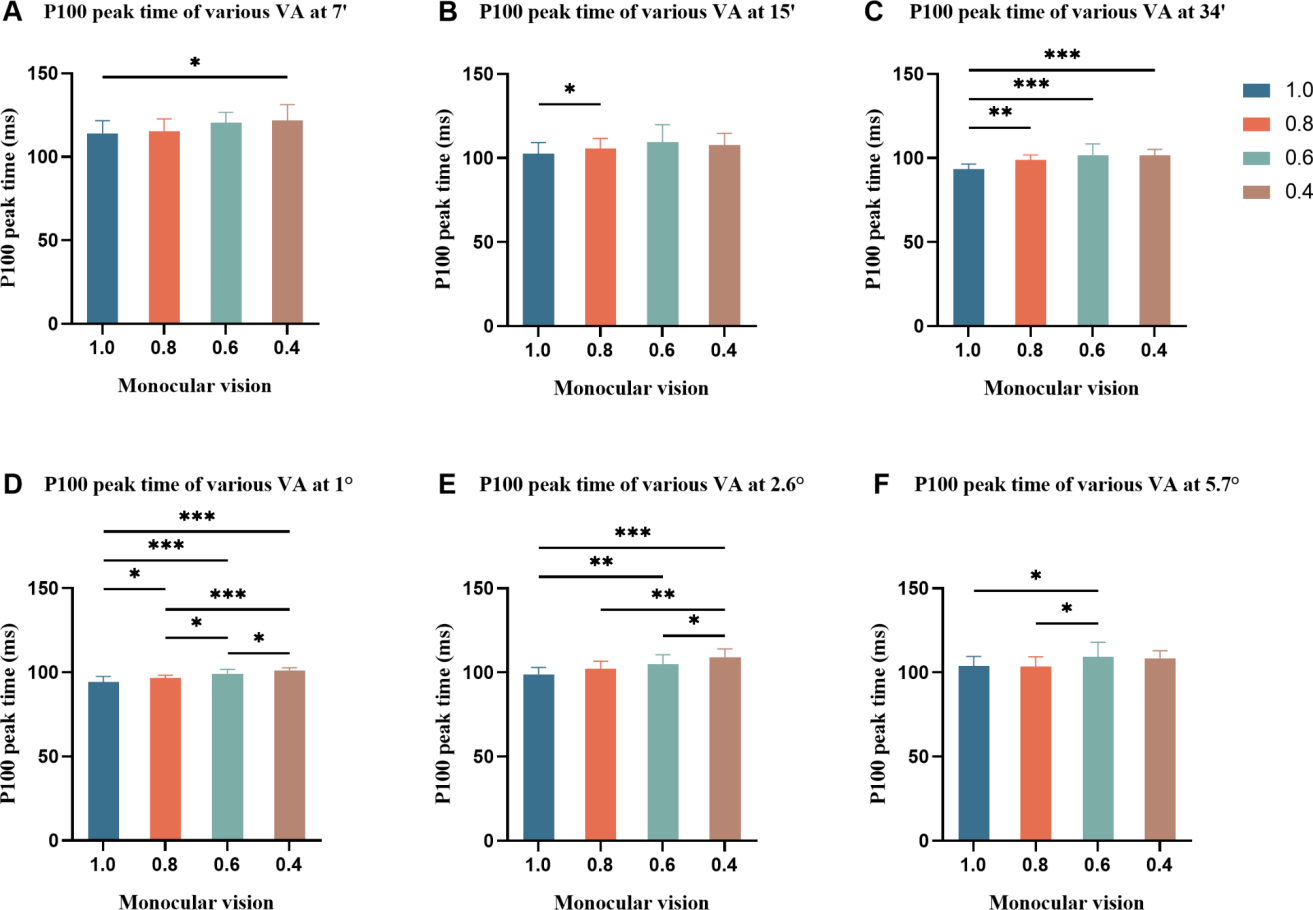
Group graph of comparison of P100 peak time of various VA at different check sizes (n = 48). ^*^P < 0.05, ^**^P < 0.01, ^***^P < 0.001. The P100 peak time of different VA at 7′ (**A**), 15′ (**B**), 34′ (**C**), 1° (**D**), 2.6° (**E**), and 5.7° (**F**)

**Fig. 3:**
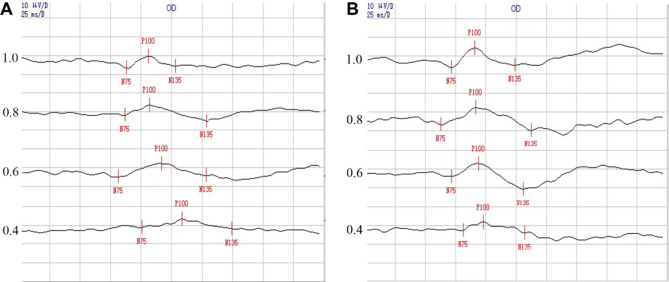
PRVEP waveforms of different vision. **A**: PRVEP waveforms at 7’ check size of different vision; **B**: PRVEP waveforms at 1° check size of different vision

There was a statistically significant difference in the main effect in P100 peak time between the different VA groups (*F* = 14.969, *P* < 0.001) and the different check sizes (*F* = 61.386, *P* < 0.001) and no statistically significant difference in the interaction between VA and check size (*F* = 0.967, *P* > 0.05). Overall, the peak time tended to increase with a decrease in VA, and the peak time of the 1.0 vision group was the shortest (101.15 ± 8.69). The peak time decreased with increasing check size, reaching the minimum value at 1° (97.73 ± 3.55). Further statistical tests revealed statistically significant differences when comparing the peak time between the vision groups with each other at 1° (all *P* < 0.05). It can be seen that compared with 1.0 vision, the P100 peak time of 0.4 vision at 7′ and 2.6°, that of 0.8 vision at 15′, those of the vision reduction groups at 34′, those of 0.6 vision at 2.6° and 5.7° were increased significantly (all *P* < 0.05) (Fig. 2). Figure 3 showed the waveforms of PRVEPs at 7′ and 1° check sizes of different VA.

### P100 amplitude versus VA at different check sizes

**Fig. 4:**
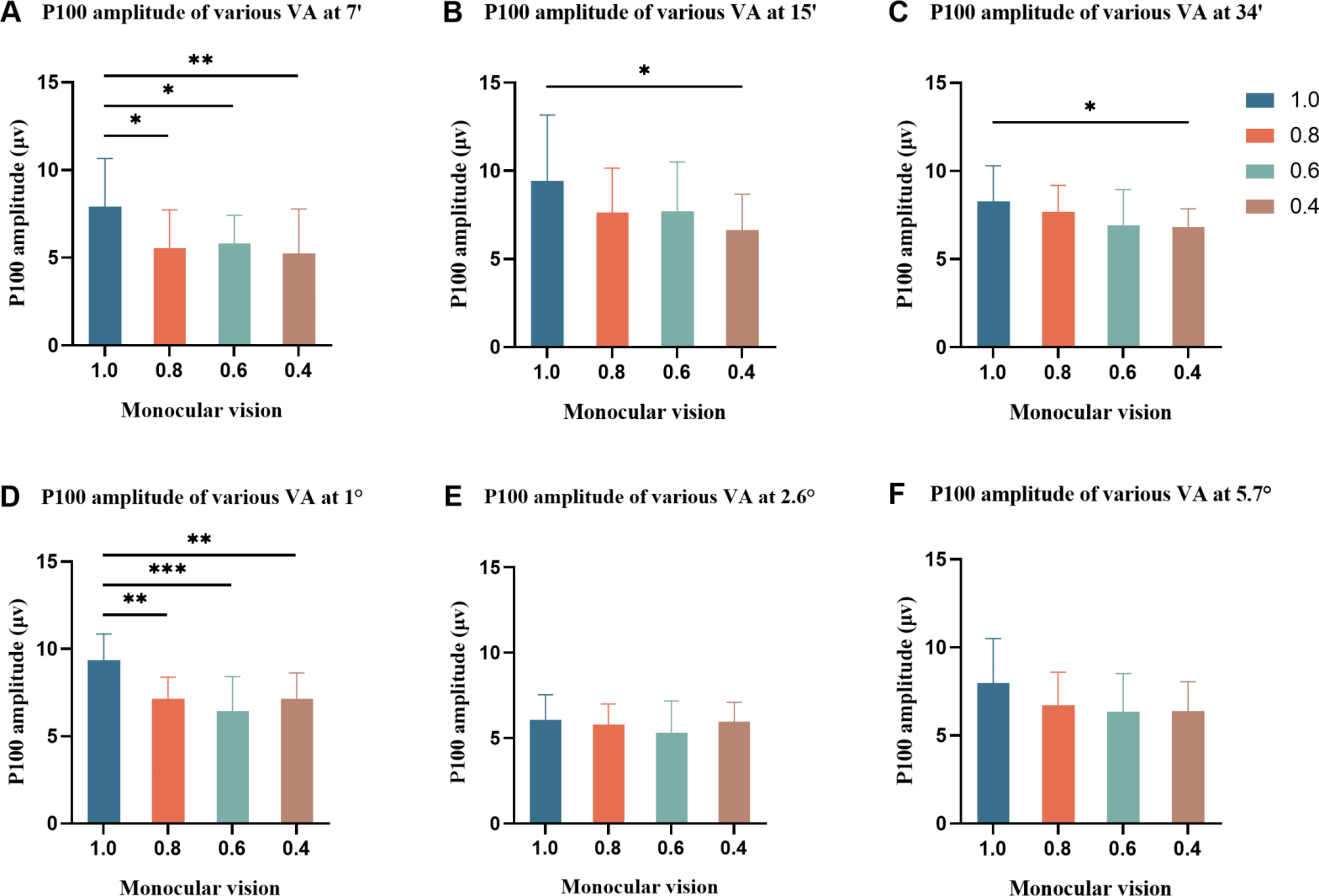
Group graph of comparison of P100 amplitude of various VA at different check sizes (n = 48). ^*^P < 0.05, ^**^P < 0.01, ^***^P < 0.001. The P100 amplitude of different VA at 7′ (**A**), 15′ (**B**), 34′ (**C**), 1° (**D**), 2.6° (**E**), and 5.7° (**F**)

There was a statistically significant main effect difference in P100 amplitude between the different VA groups (*F* = 3.153, *P* = 0.034) and the different check sizes (*F* = 27.155, *P* < 0.001) and a statistically significant difference in the interaction between VA and the check size (*F* = 1.955, *P* = 0.024). Overall, the amplitude tended to decline with a decrease in VA. The amplitude of the 1.0 vision group (7.98 ± 2.52) was the highest among all groups and the amplitude of the 15′ (7.86 ± 2.92) was the highest among all check sizes. At the 7′ and 1° check sizes, the amplitude of the vision reduction groups decreased significantly compared to that of the 1.0 vision group (all *P* < 0.05). At the 15′ and 34′ check sizes, the amplitude of the 0.4 vision group decreased significantly compared to that of the 1.0 vision group (all *P* < 0.05) (Fig. 4).

### Correlation analysis of P100 peak time and amplitude versus VA

**Fig. 5:**
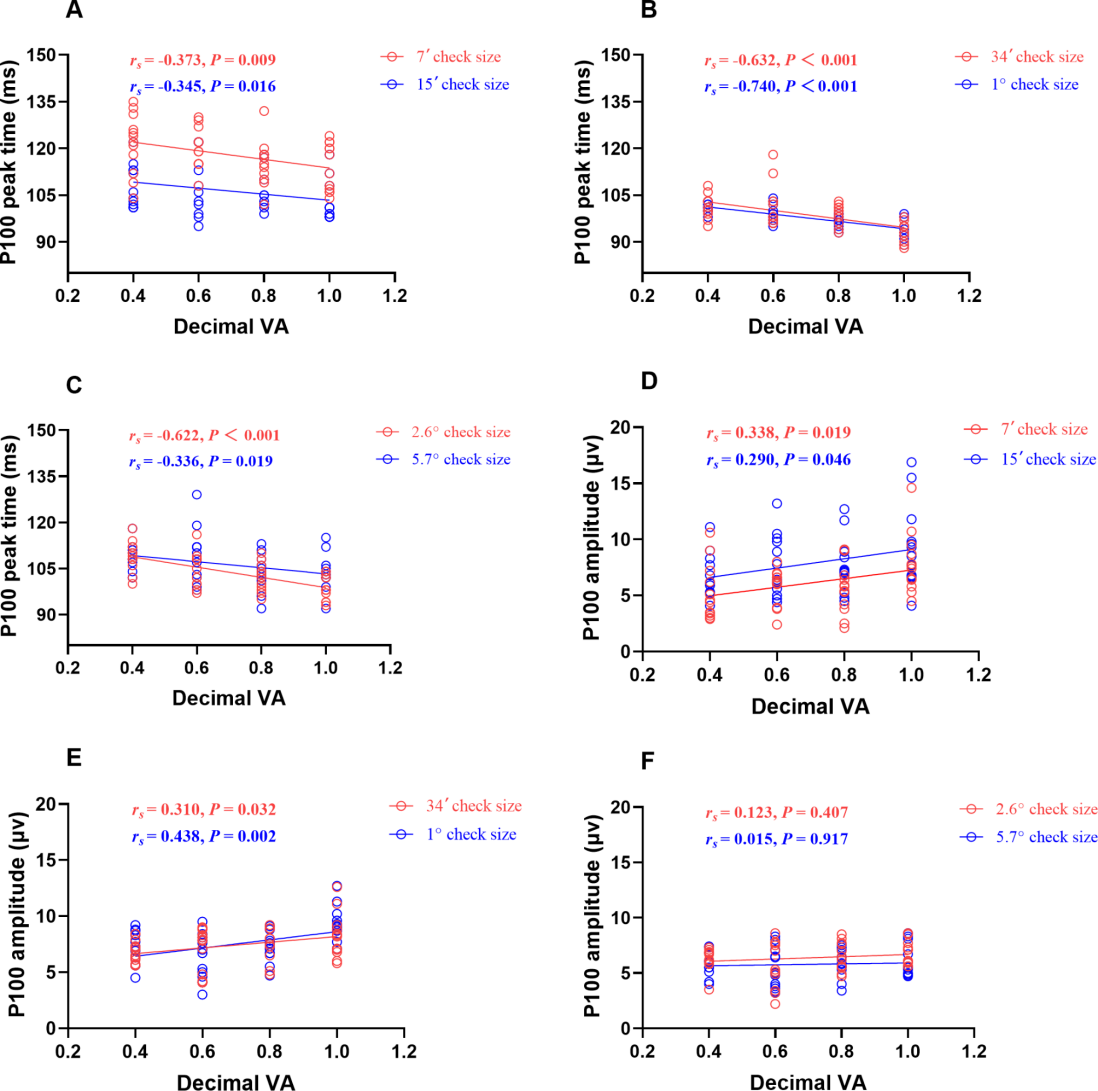
Group graph of correlation of P100 peak time and amplitude versus VA at different check sizes (n = 48). The relationships between VA and P100 peak time at 7′, 15′ (**A**), 34′, 1° (**B**), 2.6°, and 5.7° (**C**) and P100 amplitude at 7′, 15′ (**D**), 34′, 1° (**E**), 2.6°, and 5.7° (**F**). Lines represent least-squares linear model fits to the data. *r*_*s*_, Spearman rank correlation coefficient

There was a negative correlation between P100 peak time and VA at all check size (all *P* < 0.05). Namely, the higher the VA, the shorter the peak time at the same check sizes, with the highest correlation at 1° check size (*r*_*s*_ = − 0.740) (Fig. 5- A, B, C). Except for the 2.6° and 5.7° check sizes, there was a positive correlation between the P100 amplitude and VA at all check sizes (all *P* < 0.05), which means that the higher the VA, the higher the magnitude at the same check size, especially at the 1° check size (*r*_*s*_ = 0.438) (Fig. 5- D, E, F).

### Correlation analysis of CS, refractive error, wavefront aberrations and visual field versus VA

**Fig. 6:**
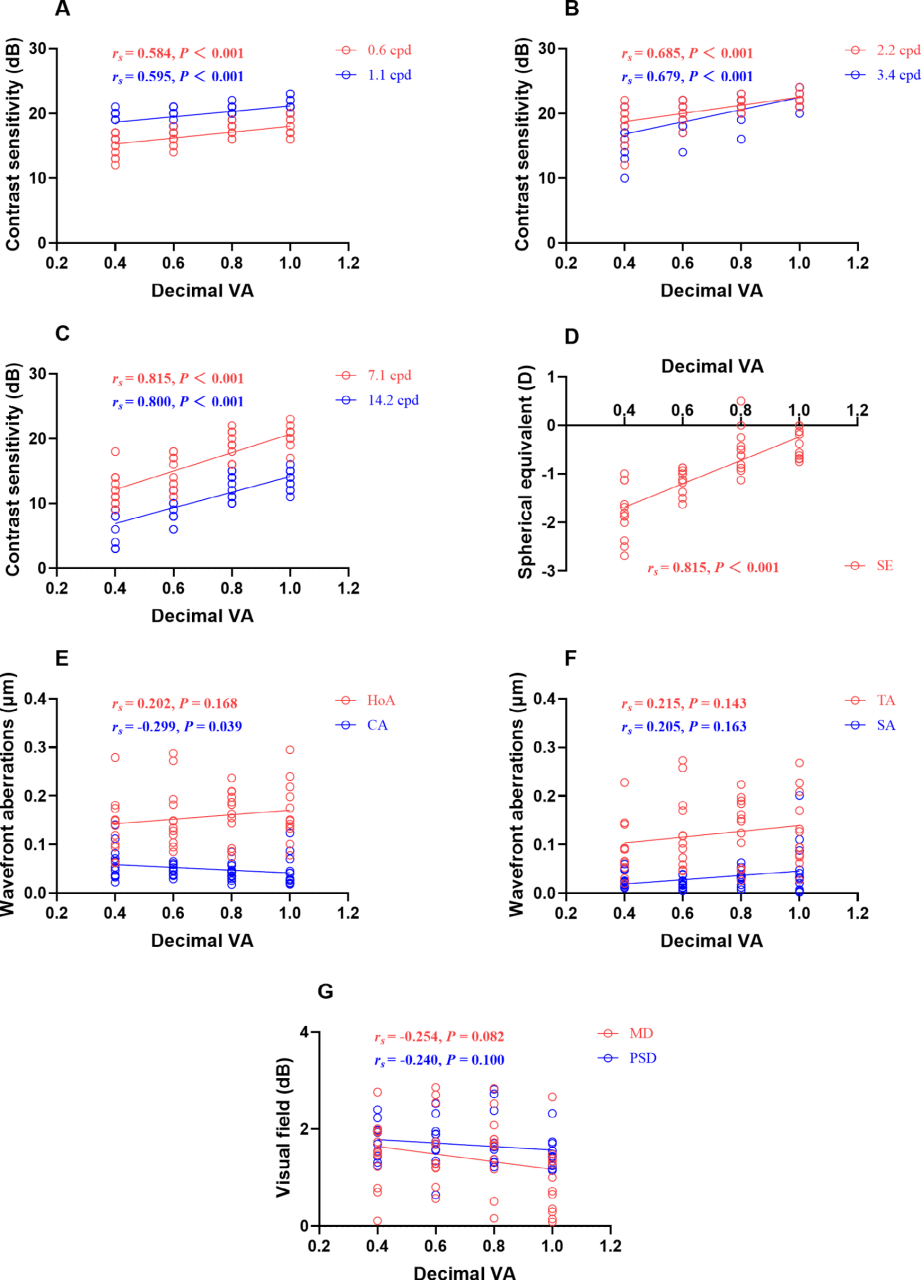
Group graph of correlation of CS, SE, wavefront aberrations and visual field versus VA (n = 48). The relationships between VA and CS of 0.6 cpd, 1.1 cpd (**A**), 2.2 cpd, 3.4 cpd (**B**), 7.1 cpd, and 14.2 cpd (**C**). The relationships between VA and SE (**D**), HoA, CA (**E**), TA, SA (**F**), MD, and PSD (**G**). Lines represent least-squares linear model fits to the data. *r*_*s*_, Spearman rank correlation coefficient. CS, contrast sensitivity; SE, spherical equivalent; VA, visual acuity; HoA, higher-order aberrations; CA, coma aberrations; TA, trefoil aberrations; SA, spherical aberrations; MD, mean deviation; PSD, pattern standard deviation

Figure 6 (A, B, C) shows correlation coefficients relating VA to CS in the spatial frequencies ranging from 0.6 cpd to 14.2 cpd. There was a positive correlation between VA and CS with all spatial frequencies (all *P* < 0.001) and high spatial frequencies (7.1 cpd and 14.2 cpd) generally had a stronger relationship to VA (*r*_*s*_ = 0.815, 0.800) than other low spatial frequencies. Figure 6- D showed that SE and VA had a statistically significant correlation (*r*_*s*_ = 0.815, *P* < 0.001). There was a negative correlation between VA and CA (*r*_*s*_ = − 0.299, *P* = 0.039), but not with HoA, TA, and SA (Fig. 6- E, F). There was no significant correlation between VA and the absolute values of both MD and PSD for the visual field (Fig. 6- G).

### Construction of classification models for objective evaluation of VA

Several classification models were constructed using machine learning algorithms such as SVM, RF, KNN, MLP, and Voting. The P100, CS, SE, and CA related to VA were input variables and binarized VA divided by different VA cutoff points were output variables. The means and standard deviations of the AUC values of the five models were obtained by training and testing with 500 replicate sampling (Table 1). For different visual acuities, the individual classifier models with the best assessment efficacy all had mean AUC values above 0.95. Similarly, the Voting classifier achieves the same assessment performance.

**Fig. 7:**
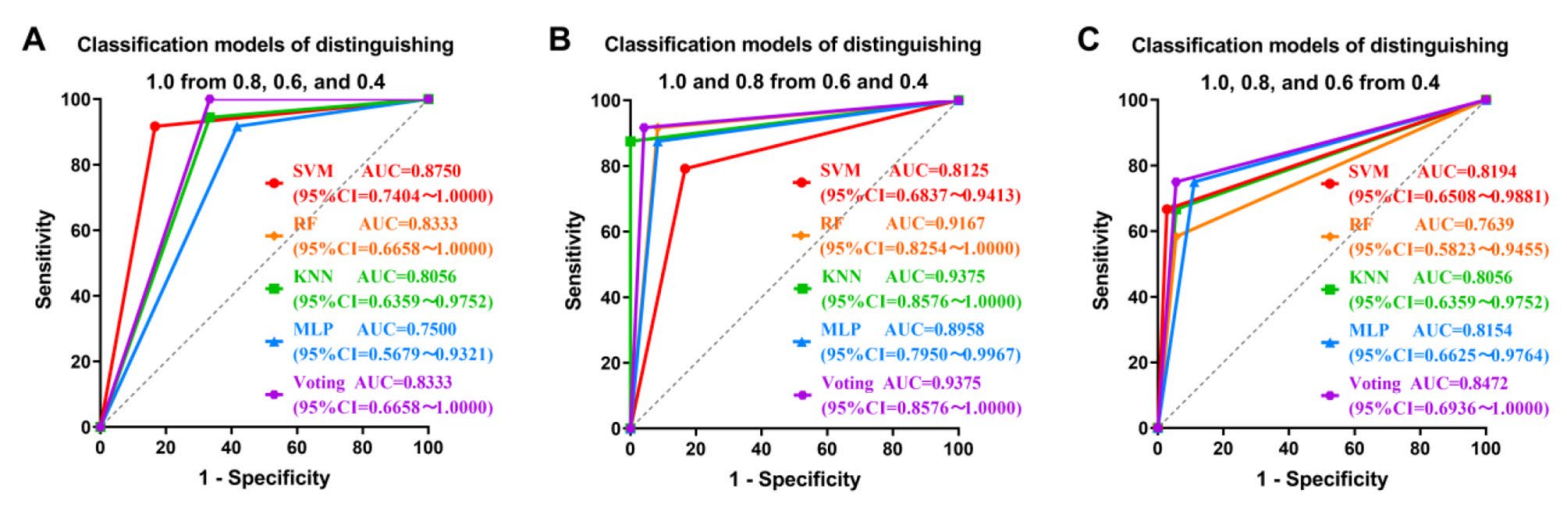
Group graph of ROC curves of ten-fold cross-validation of machine learning classification models (n = 48). The ROC curves of distinguishing 1.0 from 0.8, 0.6, and 0.4 (**A**), 1.0 and 0.8 from 0.6 and 0.4 (**B**), 1.0, 0.8, and 0.6 from 0.4 (**C**). The 95% CI of the AUC value has been demonstrated in the figures. AUC, area under the receiver operating characteristic curves; SVM, support vector machine; RF, random forest; KNN, K-nearest neighbor; MLP, multilayer perceptron

We also compared the classification performances of the ten-fold cross-validation of the five models for 48 samples (Table 2; Fig. 7). In the classification models of distinguishing between 1.0 and 0.8, 0.6, and 0.4 at the VA level, the SVM classifier performed well with an accuracy of 89.58%, sensitivity of 91.67%, specificity of 83.33%, and AUC value of 0.8750. In the classification models of distinguishing between 1.0 and 0.8 from 0.6 to 0.4 of VA, the Voting classifier performed well with an accuracy of 93.75%, sensitivity of 91.67%, specificity of 95.83%, and AUC value of 0.9375. In the classification models of distinguishing between 1.0, 0.8, and 0.6 from 0.4 at the VA level, the Voting classifier performed best with an accuracy of 89.58%, sensitivity of 75.00%, specificity of 94.44%, and AUC value of 0.8472.

**Table 1:** Comparison of AUC for 500 replicate samples of machine learning classification models for different VA objective assessment ($\stackrel{-}{x}$± s, n = 48)

Cutoff point	AUC mean value				
	SVM	RF	KNN	MLP	Voting
1.0	0.9789 ± 0.0339	0.9827 ± 0.0306	0.9125 ± 0.0671	0.9010 ± 0.0750	0.9795 ± 0.0344
0.8	0.9450 ± 0.0503	0.9891 ± 0.0187	0.9793 ± 0.0300	0.8988 ± 0.1693	0.9869 ± 0.0202
0.6	0.9640 ± 0.0478	0.9485 ± 0.0523	0.9438 ± 0.0527	0.8751 ± 0.0909	0.9558 ± 0.0504

AUC, area under the receiver operating characteristic curves; SVM, support vector machine; RF, random forest; KNN, K-nearest neighbor; MLP, multilayer perceptron.

**Table 2:** Comparison of the effectiveness of ten-fold cross-validation of machine learning classification models for different VA objective assessments (n = 48)

Cutoff point	Classification model	Accuracy	Sensitivity	Specificity	Positive predictive value	Negative predictive value
1.0	SVM	0.8958	0.9167	0.8333	0.9429	0.7692
	RF	0.9167	1.0000	0.6667	0.9000	1.0000
	KNN	0.8750	0.9444	0.6667	0.8947	0.8000
	MLP	0.8333	0.9167	0.5833	0.8684	0.7000
	Voting	0.9167	1.0000	0.6667	0.9000	1.0000
0.8	SVM	0.8125	0.7917	0.8333	0.8261	0.8000
	RF	0.9167	0.9167	0.9167	0.9167	0.9167
	KNN	0.9375	0.8750	1.0000	1.0000	0.8889
	MLP	0.8958	0.8750	0.9167	0.9130	0.8800
	Voting	0.9375	0.9167	0.9583	0.9565	0.9200
0.6	SVM	0.8958	0.6667	0.9722	0.8889	0.8974
	RF	0.8542	0.5833	0.9444	0.7778	0.8718
	KNN	0.8750	0.6667	0.9444	0.8000	0.8947
	MLP	0.8542	0.7500	0.8889	0.6923	0.9143
	Voting	0.8958	0.7500	0.9444	0.8182	0.9189

AUC, area under the receiver operating characteristic curves; SVM, support vector machine; RF, random forest; KNN, K-nearest neighbor; MLP, multilayer perceptron.

## Discussion

The results of this study showed that with the decrease of VA, the P100 peak time tended to lengthen and the P100 amplitude tended to decrease. The peak time reflects the optic nerve conduction function of the subject eye: the longer the peak time, the slower the nerve conduction speed. The amplitude reflects the number of neurons that generate electrical activity in the human eye responding to picture stimulus [22]: the lower the amplitude, the fewer the neurons stimulated. As a result, when vision decreases due to refractive error, the image projected onto the retina becomes blurred and the optic nerve pathway becomes less responsive to stimuli, eventually leading to longer peak times and decreased amplitude. The stimulus check size was “non-linear” to the peak time and amplitude. The shortest peak time was observed at 1° and the peak time increased gradually with the change of the check size. This finding was similar to those of Chen and her colleagues [11], who observed that when the VA is less than 0.2, the peak time is shortest at the 1° check size. Kurita-Tashima [23] found a curvilinear relationship between the P100 peak time and the check size in normal-vision subjects, with a minimum peak time at 35′. Steele et al. [12] studied the relationship between six check sizes from 96′ to 2′ and the amplitude and found that the maximum amplitude occurred at the 12′ check size. Consistent with their research, we also observed that the amplitude of the 15′ check size was higher than other check sizes. A possible explanation for these results may be that the edges of the stimulus pattern become blurred when the stimulus check size is small, resulting in a delay in peak time and a decrease in amplitude. Interestingly, we found that the correlations between P100 peak time, amplitude, and VA were highest at the 1° check size. It seems that greater efficacy of assessing visual acuity using peak time and amplitude of PRVEPs with 1° check size. This may be because the tests included subjects with different visual acuities and the 1° check size was moderate and appropriate for the central retinal area of these visual acuity individuals. Sun et al. [5] revealed a good correlation between VA and medium checkerboard size, especially at the 50′ check size, by examining PRVEPs to identify malingering. It has also been reported that a 15′ or 1° check size is better for studying visual-evoked potentials in hereditary optic neuropathy [24]. These differences may be partly explained by the various selection of the subject groups and the stimulus parameters. In addition, the International Society for Clinical Electrophysiology of Vision recommends 1° and 15′ as common stimulation check sizes for PRVEPs examinations [21].

The following methods have been reported to use PRVEPs to assess objective VA [25]: (1) converting the minimum check size of the recordable waveform into VA; (2) determining the spatial frequency that produces the optimal peak time and amplitude; (3) making a linear regression equation of amplitude-check size, extrapolating the amplitude to 0 µv, and taking the cutoff value; (4) evaluating the peak time and amplitude of the binocular VEP together; and (5) determining the absolute value of P100 parameters. A review study by Hamilton et al. [10] considered that direct conversion of the perspective of PRVEPs threshold stimuli to subjective VA is inaccurate. For one thing, subjective VA tests use a fixed target, whereas PRVEPs are dynamic and continuous visual stimuli. For another, the higher cognitive cortex is involved in the subjective VA tests, but the PRVEPs are only cellular activities of the primary visual cortex. Jeon et al. [26] found a significant positive correlation between P100 amplitude and VA in normal and amblyopic individuals. By plotting ROC curves, they determined that an absolute amplitude value > 5.77 µv helped distinguish between visual disability and malingering. However, due to individual variability in examining PRVEPs and the influence of various check parameters (such as pattern contrast, pattern size, average luminance, signal filtering, patient age, and pupil size), there may be some error in inferring VA from the absolute value of the P100 peak time or amplitude for a single check size. In addition, wavefront aberration, CS, refractive error, and visual field are also closely related to VA. The minimum angle of resolution (MAR) varied linearly with the magnitude of wavefront aberration [14]. Correcting wavefront aberration can improve mesopic CS and improve the quality of long-distance vision [27, 28]. Higher-order aberrations were associated with diminished visual acuity and perception in highly aberrated eyes [29]. Far visual acuity (logMAR) significantly correlates negatively with peak CS [13]. There was a correlation between the visual field mean deviation and VA in patients with glaucoma [16]. This study similarly found a correlation between CS, SE, and CA and visual acuity. Some of the discrepancies in results may be due to differences in subject populations.

Yperman et al. [30] found that the machine learning model based on visual-evoked potentials showed good predictive performance in analyzing disease progression in multiple sclerosis. Bach et al. [31] assessed objective VA with 89 machine learning algorithms based on steady-state brief-onset low-contrast checkerboard stimulation evoked potentials. They found that nearly half of the machine algorithms obtained higher agreement between subjective and objective VA than traditional heuristics and were more widely used and testable. They concluded that the machine learning approach is a useful alternative to traditional VA assessment. In contrast, we built five machine learning models based on combining the P100 values of multiple check sizes and other visual parameters (CS, SE, and CA) correlating with visual acuity to assess VA objectively. The performance of machine learning models was evaluated internally by ROC curves with the random split and the ten-fold crossover. The results of the study showed that in the internal validation results of machine learning models distinguishing different VA, the average AUC values of the 500 random split validations of the models with better evaluation performance were above 0.95 (Table 1), and the AUC values of ten-fold cross-validations were above 0.84 (Fig. 7). Both the single machine learning model and the ensemble classifier method (Voting) showed better evaluation performance. Voting exhibited evaluation performance that was superior or approximate to that of a single model. However, this study only performed an internal validation and comparison of the evaluation performances of different models. Which model has a more powerful generalization capability and which pattern performs better in objective evaluation of VA based on multiple visual parameters or a single visual parameter needs to be compared by the external validation of the model in subsequent studies. Nevertheless, the results of the internal validation of the models obtained in our current study indicated that the performance of objective assessment for VA through the parameters of P100, CS, SE, and CA was satisfactory.

Our study had several limitations that need to be considered. Firstly, the study sample size was small and only four VA points were selected. The machine learning model could not evaluate VA outside the modeling data, which means that a new VA evaluation model must be established when assessing the other VAs is needed. Secondly, the subjects in this trial were all male, and it has been shown that the standard values of the P100 peak time and amplitude of PRVEPs differ between genders [32]. Finally, our current experiment focuses on visual acuity assessment in subjects with vision loss due to refractive error, and further research is needed for visual acuity assessment in visual system disorders. Consequently, it is necessary to increase and enrich the groups and expand the range of VA to improve the availability of the test results and the reliability of the method.

## Conclusions

In conclusion, the correlations between P100 peak time, amplitude, and VA were highest at the 1° check size. There were positive correlations between CS, SE, and VA and a negative correlation between CA and VA. Furthermore, the classification models with AUC values above 0.84 were available in various VA cutoff point divisions. Machine learning classification models based on PRVEPs and other visual parameters appear to be an effective approach to the intelligent assistance of VA evaluation.

## Data Availability

The datasets used and/or analyzed during the current study are available from the corresponding author on reasonable request.

## Abbreviations

ANOVA: analysis of variance
AUC: area under the receiver operating characteristic curves
CA: coma aberrations
cpd: cycle per degree
CS: contrast sensitivity
D: degree
HoA: higher-order aberrations
KNN: K-nearest neighbor
MD: mean deviation
MLP: Multilayer perceptron
PRVEPs: pattern-reversal visual evoked potentials
PSD: pattern standard deviation
RF: Random forest
RMS: root-mean-square
ROC: receiver operating characteristic
SA: spherical aberrations
SE: spherical equivalent
SVM: Support vector machine
TA: trefoil aberrations
VA: visual acuity.
